# Mesoscopic Mapping of Ictal Neurovascular Coupling in Awake Behaving Mice Using Optical Spectroscopy and Genetically Encoded Calcium Indicators

**DOI:** 10.3389/fnins.2021.704834

**Published:** 2021-07-23

**Authors:** Fan Yang, Jing Li, Yan Song, Mingrui Zhao, James E. Niemeyer, Peijuan Luo, Dan Li, Weihong Lin, Hongtao Ma, Theodore H. Schwartz

**Affiliations:** ^1^Department of Neurology, The First Hospital of Jilin University, Changchun, China; ^2^Department of Neurological Surgery, Brain and Mind Research Institute, New York Presbyterian Hospital, Weill Cornell Medicine of Cornell University, New York, NY, United States; ^3^School of Nursing, Beihua University, Jilin City, China; ^4^Department of Radiology, The First Hospital of Jilin University, Changchun, China

**Keywords:** neurovascular coupling, awake, ictal event, mesoscopic optical imaging, mice

## Abstract

Unambiguously identifying an epileptic focus with high spatial resolution is a challenge, especially when no anatomic abnormality can be detected. Neurovascular coupling (NVC)-based brain mapping techniques are often applied in the clinic despite a poor understanding of ictal NVC mechanisms, derived primarily from recordings in anesthetized animals with limited spatial sampling of the ictal core. In this study, we used simultaneous wide-field mesoscopic imaging of GCamp6f and intrinsic optical signals (IOS) to record the neuronal and hemodynamic changes during acute ictal events in awake, behaving mice. Similar signals in isoflurane-anesthetized mice were compared to highlight the unique characteristics of the awake condition. In awake animals, seizures were more focal at the onset but more likely to propagate to the contralateral hemisphere. The HbT signal, derived from an increase in cerebral blood volume (CBV), was more intense in awake mice. As a result, the “epileptic dip” in hemoglobin oxygenation became inconsistent and unreliable as a mapping signal. Our data indicate that CBV-based imaging techniques should be more accurate than blood oxygen level dependent (BOLD)-based imaging techniques for seizure mapping in awake behaving animals.

## Introduction

Neurovascular coupling (NVC) describes the intimate relationship between neuronal activation and the resulting rise in cerebral blood flow (Kocharyan et al., 2008). NVC-based brain imaging techniques, such as functional magnetic resonance imaging (fMRI), positron emission tomography (PET), and single-photon emission computed tomography (SPECT), rely on NVC to map brain activity in humans and have been used clinically to localize epileptic foci in preparation for treatment. However, in epilepsy patients, NVC can break down or be altered as a result of chronic structural, chemical, or metabolic alteration in brain tissue (Suh et al., 2005a, 2006; Ma et al., 2009a,b, 2013; Zhao et al., 2009, 2011; Geneslaw et al., 2011). A detailed understanding of NVC during seizure activity requires techniques that can simultaneously measure both electrical and hemodynamic activity with high spatiotemporal resolution and widespread spatial sampling. Such a combination does not yet exist in clinical practice. As a result, current knowledge of NVC during seizure activity derives predominantly from animal models recorded under general anesthesia.

In prior laboratory studies, hemodynamic signals have been recorded using intrinsic optical signals (IOS) (Bahar et al., 2006; Zhao et al., 2007, 2009, 2011; Ma et al., 2009b, 2013), blood oxygen level dependent (BOLD) fMRI (Nersesyan et al., 2004b; Mäkiranta et al., 2005), near-infrared spectroscopy (NIRS) (Hoshi and Tamura, 1992), laser Doppler (Nersesyan et al., 2004a) and oxygen-sensitive electrodes (Zhao et al., 2009). Neuronal activity was derived from local field potential (LFP) or single-unit recording, both of which are severely limited by spatial sampling. Therefore, limited data exists on the spatial overlap between neuronal activity and hemodynamic changes on a mesoscopic scale. In one prior study, our group recorded simultaneous voltage-sensitive dye (VSD) and IOS in isoflurane-anesthetized rats, but not only were animals anesthetized but the VSD signal is mostly derived from the subthreshold activity, which may spread spatially far beyond the confines of the spiking cells in the ictal core.

Recently, mouse lines with genetically encoded calcium indicators (GCaMP6f) have become available for laboratory use. C57BL/6J-Tg(Thy1-GCaMP6f)GP5.5Dkim/J specifically labels pyramidal cells and the signal is derived primarily from somatic calcium influx associated with action potentials, with weaker signal resulting from subthreshold activity in the neuropil (Jercog et al., 2016). Our lab has also established methods for recording in awake-behaving animals to eliminate the known impact of anesthesia on the hemodynamic response (Gao et al., 2017; Sumiyoshi et al., 2019). In the current study, we employed simultaneous wide-field IOS, LFP, and GCaMP6f to record the neuronal and hemodynamic change during 4-AP induced ictal events in awake behaving mice. As a control, we also recorded similar data in isoflurane-anesthetized mice. IOS allows the simultaneous recording of total hemoglobin (HbT), which reflects cerebral blood volume (CBV), as well as oxy- (HbO) and deoxygenated hemoglobin (Hbr). GCaMP6f provides a sensitive detector of neuronal action potentials with fast response kinetics (Chen et al., 2013). We found that the ictal calcium signal although initially more restricted, eventually propagated more widely in awake mice often to the contralateral hemisphere. The HbT signal was also dramatically larger in awake mice, which rendered the “epileptic dip” in oxygenated hemoglobin inconsistent and a poor mapping signal. Comparing with oxygen-level-based signals, HbT provided a much better mapping signal for delineating the ictal core. Our results can be used to inform the interpretation of hemodynamic-based imaging modalities employed in the clinic to map ictal onset and propagation for seizure classification and treatment.

## Materials and Methods

All experimental procedures were approved by the Weill Cornell Medical College Animal Care and Use Committee following the National Institutes of Health guidelines. Experiments were reported in compliance with the ARRIVE guidelines. Adult C57BL/6J-Tg(Thy1-GCaMP6f)GP5.5Dkim/J mice (JacksonLab, #024276) of both sexes were employed (20–30 g, 3–6 months).

### Animal Preparation and Imaging Window Implantation

All mice were anesthetized with isoflurane in 70% N_2_: 30% O_2_, 5% induction, and 1–1.5% maintenance for the surgery. Body temperature was maintained at 37°C with a regulated heating blanket (Harvard Apparatus). The heart rate, SpO_2_, and the end-tidal carbon dioxide (EtCO_2_) were carefully monitored with a small animal capnograph (Surgivet) and were sustained throughout the experiment (heart rate: 300–450 beat/min, pO_2_ > 95%, EtCO_2_ ∼ 25–28 mmHg). The head was fixed in a stereotaxic apparatus. A ∼4 mm × 6 mm cranial window was opened and spanned the midline, between lambda and bregma. The dura was left intact. A ∼5 mm × 7 mm PDMS film (Heo et al., 2016) was carefully placed over the cranial window to ensure all exposed dura was covered. The edge of the film was fixed to the skull with surgical glue. A head plate was mounted over the film with dental cement. The mice were then individually housed in a 12:12 h light-dark cycle for recovery. Two weeks later, the mice were randomly separated into awake and anesthetized groups. For the awake imaging experiment, the mice were fixed on the imaging chamber for a 30-min training every day for three continuous days. The head plate was fixed to a clamp to immobilize the head. The mouse was placed on an air floated chamber, allowing the mouse to artificially ambulate within the chamber.

### Ictal Model and Electrophysiology

Focal injection of 4-Aminopyridine (4-AP, Sigma-Aldrich, St. Louis, MO, United States) was employed to create an acute seizure model. We screened the lowest dose that could reliably induce repeatable ictal events (>10 s induration) with reasonable intervals (>60 s). These doses differed in the awake and the isoflurane-anesthetized state, however, the dose required to elicit seizure in the awake state would not be sufficient under anesthesia whereas the dose required for such seizures under anesthesia would result in status epilepticus in the awake condition. In awake, behaving mice, focal injection of 200 nl, 2 mM 4-AP was the optimal dosage to create reproducible periodic ictal events with consistent inter-ictal intervals. The mouse was put in the head-fixed imaging system and 4-AP was injected 300–500 μm below the cortical surface through a glass microelectrode (50–100 μm tip opening), using a Nanoject II injector (Drummond Scientific, Broomall, PA, United States). The LFP was also recorded through the 4-AP electrode. The LFP was amplified (1,000×) and band-pass filtered (1–500 Hz) using a Grass amplifier, digitized *via* CED Power 1401, and recorded by a computer running Spike2 software (Cambridge Electronic Design, Cambridge, United Kingdom). Neural dynamics were recorded for 60–120 min following the 4-AP injection.

For anesthetized mice, the lowest dose that could induce reproducible periodic seizures with a consistent inter-ictal interval was a higher concentration of 200 nl, 5 mM 4-AP. Mice were anesthetized with isoflurane, 5% induction, and 1–1.5% maintenance. The head was placed in a stereotaxic apparatus. We used the same set-up as awake mice to induce seizures and record LFP.

### Wide Field Imaging

A “temporal separation” technique was employed to simultaneously image wide-field calcium and a multispectral IOS (Ma et al., 2014a, b). A CCD camera (J-MC023MGSY, Lighting Mind Inc., Changchun, China) using a tandem lens (85 mm × 50 mm) arrangement was focused 300–400 μm below the cortical surface. Three LEDs with coupled bandpass filters were employed as the illumination source, including a “blue” LED (470 ± 10 nm) for calcium imaging, a “green” LED (530 ± 10 nm), and a “red” LED (610 ± 10 nm) for IOS imaging. The illumination was directed to the cortex using optical fibers. A 510 nm long-pass filter was placed before the camera. The multispectral switching among the three LEDs was time-locked to camera frames using an Arduino board. The calcium imaging was performed every other frame and green and red IOS was performed every 2nd and 4th frame. The camera was sampling at 120 Hz, resulting in a 60 Hz imaging for calcium and 30 Hz for green and red IOS, respectively.

### Disruption of Vasodilation

In another set of experiments, dextran-conjugated fluorescein isothiocyanate (FITC-dx; #46945; Sigma-Aldrich, St. Louis, MO, United States) was injected intravenously to investigate the role of vasodilation in our imaging results. FITC-dx when illuminated with 470 nm light will disrupt local endothelial cells, thereby restricting vasodilation, while leaving neurons intact (Chen et al., 2014). In these experiments, we first injected 4-AP (2 mM, 200 nl) in head-fixed awake mice to induce ictal events. Simultaneous LFP and IOS imaging were performed to record the earlier 3–5 ictal events. The animal was then transiently anesthetized with an isoflurane-soaked cotton ball for FITC-dx injection. 50 μL of 10% FITC-dx in saline was carefully injected through the retro-orbital sinus. Isoflurane was removed after FITC-dx injection and the animal became fully awake in a short time (10s of seconds). The 4-AP injection cortex was exposed to 470 nm illumination (∼6 mW/mm^2^) for ∼7 min to disrupt the endothelial cells. Simultaneous LFP and IOS imaging were then performed for another 60 min to record ictal events. Since the excitation wavelength of FITC-dx is the same as the excitation wavelength of GCaMP6f, calcium imaging was not performed on these mice to avoid unnecessary irradiation from blue light. The experiment protocol was diagramed in Supplementary Figure 1A.

### Data Analysis

Custom-written software in MATLAB 2018A was used for data processing and statistical analysis. The pulsation artifact from the heartbeat was eliminated with an offline algorithm (Ma et al., 2004, 2014a). Briefly, an average QRS interval was obtained for each pixel in each trial. The peaks of R waves were obtained from ECG. An averaged pulsation artifact was obtained by an R wave-triggered average. This averaged pulsation artifact was repeatedly subtracted from each heartbeat cycle of the original data. In order to increase the signal-to-noise ratio, imaging data were convolved with a spatial Gaussian kernel (σ = 3 pixels).

A modified Beer-Lambert law, a path length correction factor, was used to calculate the concentration of deoxygenated hemoglobin (Hbr), oxygenated hemoglobin (HbO), and total hemoglobin (HbT) change using 530 nm and 610 nm IOS data (Sheth et al., 2004). A 2 Hz Butterworth low pass filter was applied to the hemodynamic signal to reduce high-frequency noise.

For calcium signal processing, the functional hemodynamic artifact was separated using the following equation (Kramer and Pearlstein, 1979; Ma et al., 2016):

*F*_*True*_(t)/*F*_*True*_(t_0_) = [F(t)/F(t_0_)]/[I(t)/I(t_0_)]

where *F*_*True*_ is the calcium fluoresce intensity absenting hemodynamic artifact, *F* is the recorded calcium fluorescence intensity, *I* is the recorded IOS signal at 530 nm, and t_0_ is the time point for baseline.

In order to calculate the spatial extent in calcium signal and IOS, a modified Chen-Bee method was employed (Chen-BeeKwon et al., 1996; Berwick et al., 2008). Briefly, 30% of the maximal amplitude of the optical signal measured from the 4-AP injection site (ictal focus) was selected as a threshold. All pixels with an amplitude above the threshold were considered active pixels. The area of spread was calculated by multiplying the number of active pixels by the area of each pixel (29.34 μm × 29.34 μm).

## Results

**FIGURE 1:**
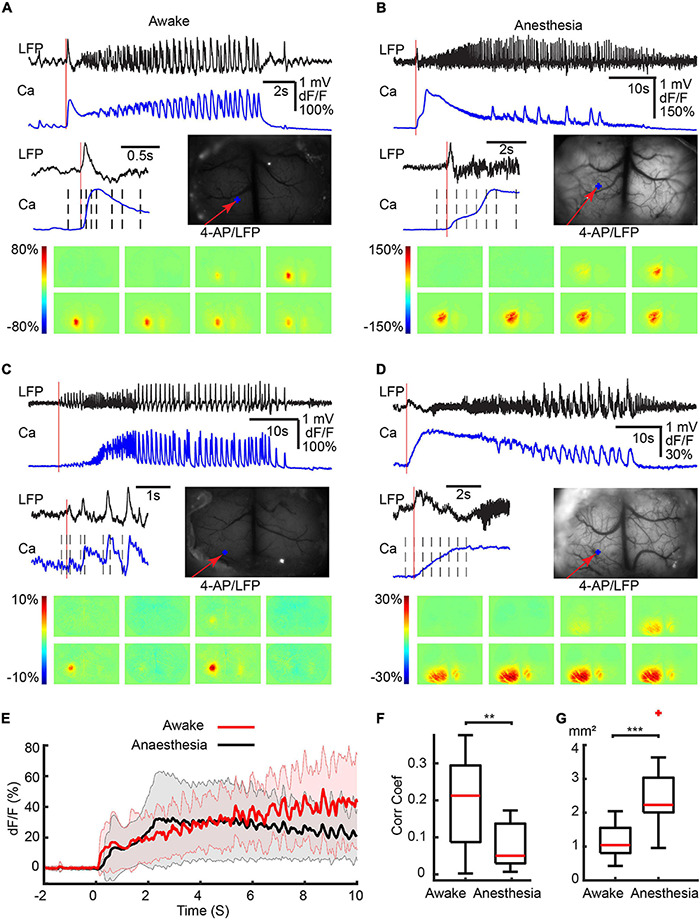
Different initiation patterns of ictal events in awake and isoflurane-anesthetized animals. Calcium imaging of different ictal initiated patterns in four separate animals. **(A)** awake with a first spike. **(B)** Anesthetized with a spike. **(C)** Awake without a first spike. **(D)** Anesthetized without a first spike. For each panel, the top shows the LFP and calcium traces recorded at the 4-AP injection site. An enlarged view of the initiation section and the field of view are shown in the middle. The vertical red line indicates the onset of the ictal event. The black dashed lines indicate the time points from which the activity maps are shown at the bottom. The red arrow shows the 4-AP injection/LFP recording electrode. The blue dots show the location where the calcium trace is recorded. **(E)** The average calcium trace was recorded from the 4-AP injection site. The solid line and the shaded area showed the mean ± SD of the averaged calcium trace (*n* = 17 seizures in eight awake animals; *n* = 14 seizures in seven anesthetized animals). Note the progressive increase and shorter decay, higher temporal resolution in the calcium signal in awake animals compared with the slow decrement low temporal resolution in the anesthetized animals. **(F)** Box plot of the correlation coefficient between the LFP and calcium traces in awake (*n* = 17 seizures in eight awake animals) and anesthetized (*n* = 14 seizures in seven animals) mice. **(G)** Box plot of the area of calcium signal involved in the initiation of ictal events in awake (*n* = 17 seizures in eight animals) compared with anesthetized (*n* = 14 seizures in seven animals) mice. ***p < 0.001 and **p < 0.01.

Ictal events were recorded with LFP electrodes from both awake and isoflurane-anesthetized mice and the waveforms were similar in morphology (Figure 1). Events began either with or without an initial spike, followed by low voltage fast activity (LVFA) and then periodic spike-and-waves discharges (Figure 1). The average duration of ictal events was not significantly different between awake (43.51 ± 27.76 s, 17 seizures; eight animals) and isoflurane-anesthetized mice (49.81 ± 21.60 s; 14 seizures; seven animals; *p* = 0.602, two-tailed-unpaired *t*-test). Awake events occurred slightly more frequently, with a periodicity of 0.279 ± 0.204 events/min compared with 0.091 ± 0.015 events/min in anesthetized animals (*p* = 0.042, two-tailed-unpaired *t*-test).

### Calcium Imaging of Ictal Onset in Awake and Anesthetized Animals

The majority of ictal events (11/17: awake and 10/14: anesthetized) were initiated with an obvious initial large spike (Figures 1A,B). The remaining ictal events (6/17: awake and 4/14: anesthetized) were initiated without an obvious initial spike (Figures 1C,D). For the ictal events that began with an initial spike, the calcium imaging differed between the awake and anesthetized mice. In awake mice, the initial spike showed a rapid increase in calcium amplitude, which reached its peak at 0.318 ± 0.081 s (Figure 1A). In anesthetized mice, on the other hand, the calcium signal rose more slowly, peaking at 1.370 ± 0.816 s (*p* < 0.001, two-tailed-unpaired *t*-test; Figure 1B). For the ictal events that began without an initial spike, in awake mice, the calcium signal progressively increased during the evolution of the seizure (Figure 1C), while in anesthetized mice the calcium signal slowly decreased from its early peak (Figure 1D).

Examining an average of the calcium signal recorded during the first 10 s of all ictal events, in awake mice, the calcium amplitude slowly increased and didn’t reach the peak amplitude within 10 s. While in anesthetized mice, the calcium reached a peak amplitude at 2.728 s after onset then gradually decreased (Figure 1E). The calcium signal in awake animals also revealed rapid signal fluctuations corresponding with the underlying fluctuations in the LFP. In the anesthetized animals, the calcium signal response to rapid fluctuations in the LFP was blunted by the anesthesia (Figure 1). In awake mice, there was a higher correlation between the calcium and LFP signals (0.191 ± 0.124) compared with the anesthetized animals where the correlation was much lower (0.072 ± 0.057, *p* = 0.0031, two-tailed-unpaired *t*-test; Figure 1F), likely caused by the presence of higher frequencies in the anesthetized state (Supplementary Figure 2) and the inherent limitations in the response times of the calcium signal. Under anesthesia, the area of the calcium signal at onset was also larger. In awake mice, ictal onset involved a more limited region in both types of ictal events than in anesthetized mice (Figures 1A–1D). On average, 1.19 ± 0.47 mm^2^ of the cortical area was recruited in awake mice, which was significantly smaller than anesthetized mice (2.61 ± 0.75 mm^2^, Figure 1F, *p* < 0.001, two-tailed-unpaired *t*-test).

### Ipsilateral Propagation of Calcium and Hemodynamic Signals

**FIGURE 2:**
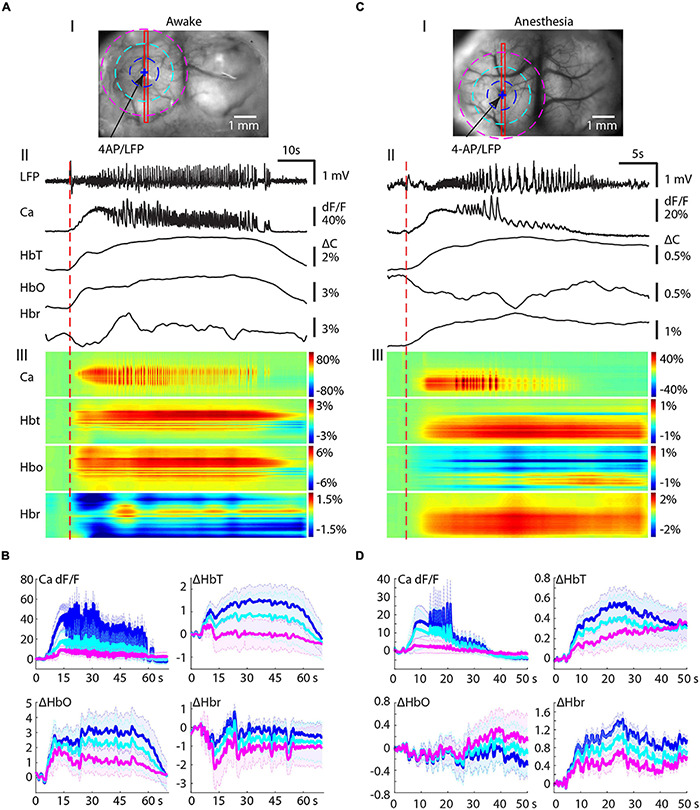
Ipsilateral propagation of calcium and hemodynamic signals in awake and isoflurane-anesthetized mice. **(A)** Ipsilateral propagation of ictal event in an awake mouse. **(A-I)** The field of view. The blue arrow indicates the 4-AP/LFP electrode. A blue + indicates a pixel PiOI from within the 4-AP injection site from which the signals are shown in panel **(A-II)**. A red rectangle box shows a linear region of interest (LiROI) from which the calcium and hemodynamic signal are shown in panel **(A-III)**. Three dotted circles indicate three ring-RiOIs from which the averaged calcium signals are shown in panel **(B)**, each corresponding to the different color of the ring. The diameters of each RiROI are 0.7, 1.4, and 2.1 mm, respectively. **(A-II)** The LFP, calcium, and hemodynamic traces from the PiROI in the 4-AP injection site. **(A-III)** The calcium and hemodynamic dynamics from the LiROI. **(B)** The average calcium and hemodynamic traces from three RiOIs. **(C)** Ipsilateral propagation of ictal events in anesthetized mice. The arrangement of panel **(C)** is the same as in panel **(A)**. **(D)** The average calcium and hemodynamic traces from the three RiOIs.

We then investigated the build-up and propagation of the calcium and the hemodynamic signals during ictal evolution within the ipsilateral cortex. In awake mice, the calcium signal reached its maximal area ∼10 s after the seizure onset and then slowly decreased until seizure termination (Figure 2A). The hemodynamic changes, on the other hand, showed a different propagation pattern. HbT increased in a slow, monomorphic wave that peaked near the termination of the ictal event and outlasted both the LFP and calcium signals. The HbT signal propagated to its maximal area earlier than the calcium signal and remained in a similar large area until seizure termination. The waveform of HbO was similar to the HbT signal but the spatial distribution was broader than HbT (Figure 2A). The Hbr signal, on the other hand, showed a more compact but inconsistent spatiotemporal dynamic. Hbr increases were observed in 8/17 ictal events and decreases were observed in the other nine events. Hbr increases correspond with a dip in oxygenation that was neither consistent nor reliable in awake animals. In surrounding areas, on the other hand, Hbr decreases were recorded during all awake events (Figure 2A).

We further characterized the spatial distribution of the signals with concentric rings of interest (RiOI). Ring diameters were set at 0.7, 1.4, and 2.1 mm (blue, light blue, and purple, respectively, in Figure 2B). The averaged signals in the center RiOI reflected an increase in calcium, HbT, and HbO. However, the average Hbr in the center RiOI fluctuated during the seizure. In the surrounding 1.4–2.1 mm RiOIs, the averaged calcium, and HbT traces remained close to the baseline, indicating restricted spread. However, the HbO signal was above baseline and the Hbr signal was far below baseline, indicating hyperoxygenation in the surround in awake animals. Our data indicate that the increases in perfusion accompanying seizures in the awake animal provide sufficient oxygenation so the focus is only slightly and occasionally hypoxic and the surround areas are hyperperfused and have an overabundance of oxygen. Moreover, the inconsistency of the Hbr signal indicates it may not be an ideal signal source for mapping seizures in awake animals.

In isoflurane-anesthetized animals, NVC was altered with an overall blunting in the amplitude of all signals (Figures 2C,D). The HbT signal was observed in a similar spatial distribution as the calcium signal (Figure 2C) but with a much longer duration. On average, HbT outlasted the ictal event by 39.88 ± 28.38 s in anesthetized animals compared with 27.05 ± 13.93 s in awake animals (*p* = 0.120, two-tailed-unpaired *t*-test). The HbO and Hbr signals were even more altered by anesthesia. In contrast to awake cases, where HbO increased, anesthesia resulted in a dramatic increase in Hbr, with a much longer duration (Figure 2C). The HbO signal in anesthetized mice showed similar spatial distribution as the Hbr signal in awake mice. In the seizure focus, HbO both increased (10/14 seizures) and decreased (4/14 seizures). The same concentric ring analysis confirmed seizure-induced global increases in Hbr and HbT signals but a fluctuation HbO signal (Figure 2D).

Our data show that in both awake and anesthetized mice, seizures trigger an increase in blood supply to the seizure focus. In awake animals, this provides an adequate supply of oxygen to the seizure focus and an oversupply to the surround. In anesthetized mice, the increased blood supply is insufficient to supply adequate oxygen to the seizure focus and the surround resulting in a longer and more consistent “epileptic dip” in tissue oxygenation (Bahar et al., 2006).

### The Amplitude of the Calcium and Hemodynamic Signals in the Ipsilateral Cortex

**FIGURE 3:**
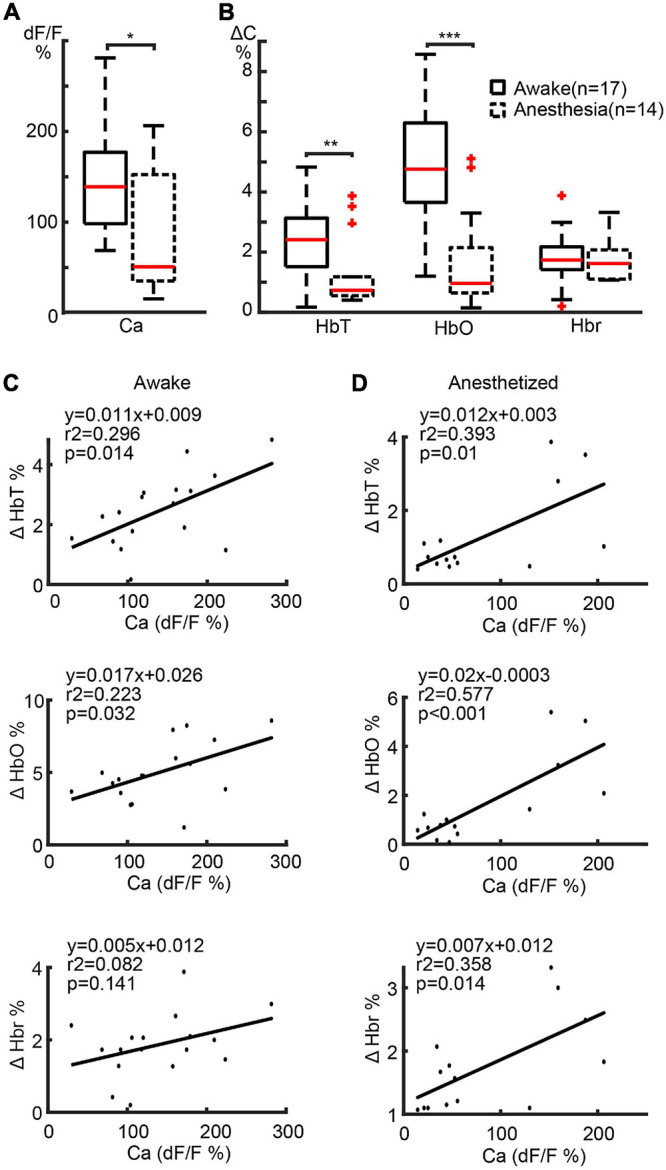
Amplitude correlation between calcium and hemodynamic signals in ipsilateral cortex. **(A)** Boxplot of maximal calcium amplitude in awake and anesthetized mice. **(B)** Box plot of maximal hemodynamic amplitude in awake and anesthetized mice. ***p < 0.001, **p < 0.01, and *p < 0.05. **(C,D)** Linear regression of maximal calcium and hemodynamic amplitude in awake and anesthetized mice, respectively. The regression equations, *r*^2^, and *p*-values are labeled in the plots.

The maximal amplitudes of the calcium and hemodynamic signals during the ictal event were examined throughout the seizure. The maximal amplitude of the calcium signals in awake mice (139.06 ± 62.02%; *n* = 17 seizures; eight mice) was significantly higher than in anesthetized mice (83.34 ± 65.27%; *n* = 14 seizures; seven mice) (*p* = 0.0257, two-tailed-unpaired *t*-test) (Figure 3A), indicating a respective increase in bursts of action potentials in the focus of awake mice compared with anesthetized mice. This data represents power changes during the evolution of the entire ictal event and should not be confused with the higher calcium signal in the first 10 s of the seizure for the animals under anesthesia shown in Figure 1. The amplitudes of the HbT and HbO signals in awake mice (2.45 ± 1.18%, 4.98 ± 2.01%, respectively) were also significantly higher than those recorded from the anesthetized mice (1.29 ± 1.14% in HbT and 1.63 ± 1.67% in HbO; *p* = 0.0122 in HbT, *p* < 0.001 in HbO, two-tailed-unpaired *t*-test; Figure 3B). However, Hbr amplitude was similar in both awake (1.87 ± 0.85%) and anesthetized (1.75 ± 0.71%) mice (*p* = 0.6893, two-tailed-unpaired *t*-test).

We further determined the relationship between the amplitudes of the calcium and hemodynamic signals using a linear regression model. In awake mice, a highly positive correlation was found between calcium and HbT (*r*^2^ = 0.296, *p* = 0.014) and HbO (*r*^2^ = 0.223, *p* = 0.032) signals (Figure 3C). The linear correlation between calcium and Hbr amplitude, on the other hand, was weaker (*r*^2^ = 0.082, *p* = 0.141) (Figure 3C). In anaesthetized mice, a highly positive correlation was found between calcium and all hemodynamic components (HbT: *r*^2^ = 0.393, *p* = 0.010; HbO: *r*^2^ = 0.577, *p* < 0.001; Hbr: *r*^2^ = 0.358, *p* = 0.014) (Figure 3D).

Our data indicate that NVC was maintained in both awake and anesthetized conditions. However, awake seizures elicited a greater blood supply to the seizure focus than anesthetized seizures. In awake mice, the increased blood supply was adequate to meet the metabolic demand leading to a greater increase in HbO and a variable but lower increase in Hbr. In anesthetized mice, the increased blood supply was not adequate and resulted in a higher level of Hbr than HbO, indicating an insufficient oxygen supply to the seizure focus causing a more prolonged “epileptic dip.”

### Contralateral Hemisphere Recruitment in Awake and Anesthetized Mice

**FIGURE 4:**
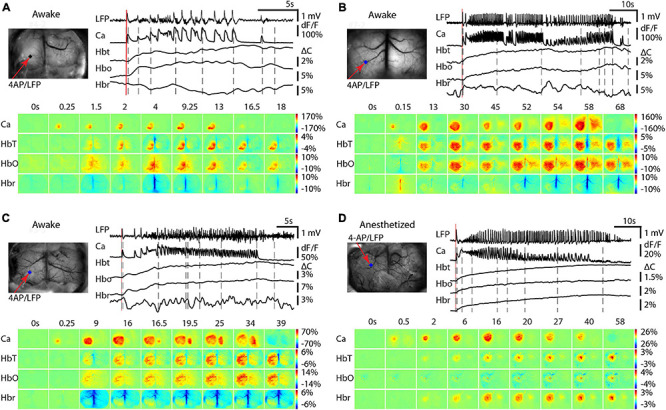
Contralateral propagation of calcium and hemodynamic signals in awake and isoflurane-anesthetized mice. In awake mice, three different propagation patterns were observed. **(A)** Calcium and hemodynamic signals remain ipsilateral. **(B)** Calcium and hemodynamic signals propagate to the contralateral hemisphere by jumping to the contralateral homotopic area. **(C)** Calcium and hemodynamic signals propagate to the contralateral hemisphere smoothly crossing the midline. **(D)** In anesthetized mice, the calcium and hemodynamic signals remained predominantly ipsilateral Small hemodynamic signals were recorded contralaterally. For each subpanel, the top left is the field of view. The red arrow shows the 4-AP/LFP electrode and the blue dot shows the POI from the 4-AP injection site. Top right, the LFP, calcium, and hemodynamic traces from the POI. Bottom, the spatiotemporal dynamics of the calcium and hemodynamic signals.

We then investigated the impact of anesthesia on the recruitment of the contralateral hemisphere for both calcium and hemodynamic signals. In awake mice, three different calcium propagation patterns were observed. In 9/17 seizures, the calcium signal remained ipsilateral with a minimal hemodynamic signal in the contralateral cortex (Figure 4A). As previously described, the Hbr signal was inconsistent (Figure 2). In the remaining 8/17 seizures, the calcium signal spread to the contralateral cortex. Two different cross-hemisphere patterns were observed. In 6/8 seizures, the seizure activity first propagated throughout the ipsilateral cortex and then jumped to the homotopic location to the 4-AP injection site in the contralateral hemisphere, presumably through commissural fibers (Figure 4B). In the remaining two events, the seizure first propagated throughout the ipsilateral cortex and then contiguously propagated to the contralateral cortex, as if spreading from one cingulate gyrus to the contralateral cingulate gyrus (Figure 4C). In the events that propagated bilaterally, the HbT and HbO signals showed an obvious increase in the similar area as calcium signal in the contralateral cortex, but no obvious Hbr change could be detected in the contralateral cortex.

In isoflurane-anesthetized mice (*n* = 14 seizures; seven mice), however, all seizures remained unilateral demonstrating uniquely ipsilateral propagation (Figure 4D). In the contralateral cortex, very low amplitude increases in HbT and Hbr were sometimes recorded.

### Spatial Correlation Between Calcium and Hemodynamics Signals in the Ipsilateral Cortex

**FIGURE 5:**
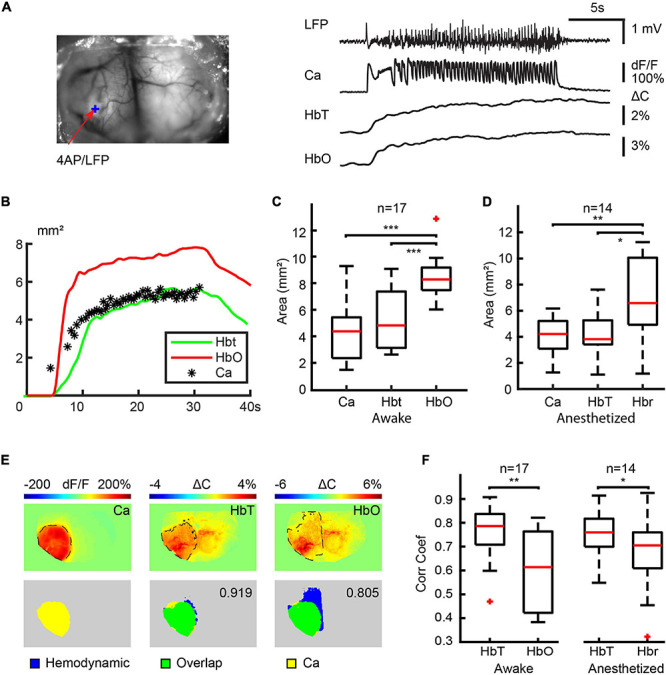
Spatial correlation between calcium and hemodynamic signals in ipsilateral cortex. **(A)** The field of view and the traces of LFP, calcium, HbT, and HbO traces from the 4-AP injection site. **(B)** The area of change during the ictal event. Note: The areas of the HbT signal more accurately map the calcium area than the HbO signal. **(C)** Box plot of a maximal area of calcium, HbT, and HbO signals in awake mice. Note: The HbT and calcium areas are not significantly different. **(D)** Box plot of maximal calcium, HbT, and Hbr signal in anesthetized mice. Note: The HbT and calcium areas are not significantly different. **(E)** Example of the spatial overlap between maximal calcium and hemodynamic areas. The top rows show the maximal area of the different signals. The dashed black line indicates the maximal area in the ipsilateral cortex defined with a modified Chen-Bee method. Bottom row: the spatial overlap between maximal calcium and hemodynamic area. The 2D correlation coefficient between calcium and the hemodynamic area is labeled. **(F)** The boxplot of the correlation coefficient between calcium and hemodynamic areas in awake and anesthetized mice. ***p < 0.001, **p < 0.01, and *p < 0.05.

In order to determine the impact of anesthesia on the spatial specificity of NVC, we further quantified the spatial correlation between the calcium and hemodynamic signal during ictal events in both anesthetized and awake animals. Having already demonstrated that the awake Hbr and anesthetized HbO signals are inconsistent, we considered they were not reasonable candidates for seizure mapping, and thus excluded them from this next set of experiments. Moreover, since ictal events displayed different degrees of contralateral propagation, we only compared the spatial correlation between the different signals on the ipsilateral side. Figure 5 shows an example of the spatial spread of the calcium and hemodynamic signals in the ipsilateral cortex in an awake mouse. Both hemodynamic and calcium signals propagated quickly after ictal onset and remain localized to a similar area until seizure termination. The spatial propagation of the HbT signal closely approximated the calcium signal for the duration of the seizure, but the HbO signal propagated to a larger area (Figure 5C). One-way ANOVA test and Tukey-Kramer *post hoc* analysis indicated that the maximal area of calcium (4.26 ± 1.98 mm^2^), HbO (8.51 ± 1.55 mm^2^), and HbT (5.35 ± 2.18 mm^2^) were significantly different (*P* < 0.001, *f* = 21.34, degree of freedom = 50). The HbO area was significantly larger than both calcium (*P* < 0.001) and HbT (*P* < 0.001), however, the maximal area of calcium and HbT signals were not significantly different from each other (*P* = 0.240). We also employed a one-way ANOVA test and Tukey-Kramer *post hoc* analysis to compare the spatial distribution of the HbT, Hbr, and calcium signals in anesthetized mice (Figure 5D). The maximal area of the calcium (4.10 ± 1.42 mm^2^), HbT (4.40 ± 1.74 mm^2^), and Hbr (6.79 ± 2.89 mm^2^) signals were significantly different from each other (*P* = 0.0042, *f* = 6.33, degree of freedom = 41). The Hbr area was significantly larger than both calcium (*P* = 0.0067) and HbT (*P* = 0.017) areas. The maximal area of the calcium and HbT signals were not significantly different from each other (*P* = 0.932). We then performed a 2-dimensional cross-correlation between the calcium area and the area of each of the hemodynamic signals (Figure 5F). In awake mice, the correlation coefficient between areas of the HbT and calcium signals (0.76 ± 0.11) was significantly higher than the HbO signal (0.61 ± 0.16) (*p* = 0.0011, two-tailed-paired *t*-test). Similarly, in anesthetized mice, the correlation coefficient between the areas of the HbT and calcium signals (0.75 ± 0.10) was also higher than the Hbr signal (0.67 ± 0.15) (*p* = 0.0129, two-tailed-paired *t*-test). Our data indicated that blood volume-based HbT signal provides a more accurate representation of the spatial spread of neuronal activity than BOLD signals, which are based mostly on changes in HbO and Hbr in both awake and anesthetized mice.

### Disruption of Vasodilation Mimics the Effect of Anesthesia

**FIGURE 6:**
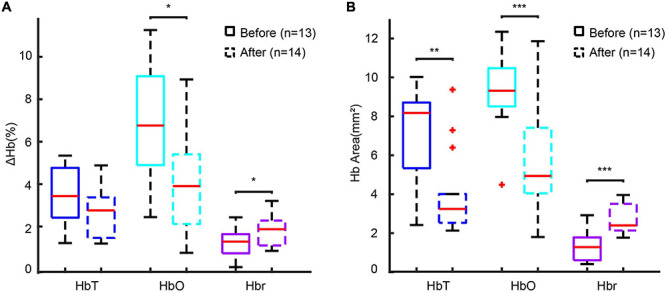
Disruption of endothelial reactivity recapitulates the effect of anesthesia. **(A)** Box plot of hemodynamic amplitude change-induced before and after endothelial disruption with photo-activation of FITC-dx. **(B)** Box plot of the hemodynamic area before and after endothelial disruption with photo-activation of FITC-dx. ***p < 0.001, **p < 0.01, and *p < 0.05.

We hypothesized that the anesthesia-induced blunting of the amplitude of the HbT response was the primary cause of the increase in Hbr, which was not found in the awake condition. We conjectured that a similar effect would be found with pharmacological blunting of the vascular response in awake animals. To test this theory, we disrupted the endothelia of the vasculature in the superficial layer of the cortex using a previously described method of photo-activation of FITC-dx in awake mice. After disruption of the endothelia, the amplitude of the HbT reduced only slightly from 3.55 ± 1.36% to 2.74 ± 1.08% (*p* = 0.1092, two-tailed-unpaired *t*-test). The HbO amplitude, however, significantly reduced from 6.88 ± 2.79% to 4.22 ± 2.13% (*p* = 0.0123, two-tailed-unpaired *t*-test). The Hbr signal, on the other hand, significantly increased from 1.40 ± 0.64% to 1.95 ± 0.68% (*p* = 0.0475, two-tailed-unpaired *t*-test) (Figure 6A). A similar change was also observed in the spatial propagation of hemodynamic signals. A significant decrease in the HbT area was detected, which reduced from 6.92 ± 2.36 mm^2^ to 4.03 ± 2.07 mm^2^ (*p* = 0.0032, two-tailed-unpaired *t*-test). The HbO area decreased from 9.34 ± 1.82 mm^2^ to 5.55 ± 2.77 mm^2^ (*p* < 0.001, two-tailed-unpaired *t*-test), and the Hbr area increased from 1.32 ± 0.70 mm^2^ to 2.65 ± 0.72 mm^2^ (*p* < 0.001, two-tailed-unpaired *t*-test) (Figure 6B). In summary, although the pharmacologic disruption of the endothelia was not a perfect recapitulation of the anesthetic effect, the decrease in HbT caused a similar under-perfusion of the hypermetabolic focus resulting in a decrease in blood oxygen level (decrease in HbO and increase in Hbr), which mimicked the influence of anesthesia. Thus, isoflurane anesthesia may act in part by blunting the functional hyperemic response resulting in a partial neurovascular uncoupling and a more profound ictal ischemia.

## Discussion

In this study, we show that in awake behaving mice, the ictal mapping signal derived from cerebral blood volume, or HbT, provides the most reliable signal for mapping both the ictal onset and spread. The previously identified “epileptic dip” is much less consistent in awake animals, and may be an artifact of an isoflurane-induced blunting of the hemodynamic response. These results are both encouraging for the development of new techniques that can provide high-resolution imaging of blood flow and blood volume for mapping of ictal onsets in humans while providing cautionary advice on interpreting oxygenation changes such as the BOLD signal as a less reliable tool for ictal mapping.

### Ictal Onset and Propagation in Awake Behaving Animals

Traditional ictal mapping using electrodes provides high temporal resolution recording of summated postsynaptic activity with limited spatial sampling (Kirschstein and Kohling, 2009; Nelson and Pouget, 2010; Herreras, 2016). Wide-field calcium imaging overcomes the spatial sampling limitations of electrodes and records signals derived primarily from somatic calcium influx associated with action potentials in specified neurons (Jercog et al., 2016). GCamp6f in pyramidal cells provides faster kinetics required for ictal mapping with fluorescence signals derived primarily from action potentials. The calcium signal can thereby provide spatial information on the neurons that participate in the ictal core at its onset and during propagation. Although similar LFP waveforms were presented in awake and anesthetized mice, spatial recruitment differed during initiation and propagation. The cortical area involved in the initiation process in awake mice was smaller than in the anesthetized mice (Figure 1G). The most likely explanation would be that isoflurane exerts its inhibitory effects in the central nervous system mainly *via* potentiation of GABA-A receptors, which causes an influx of chloride ions into postsynaptic neurons with subsequent hyperpolarization of the cell membrane (Ito et al., 1998). Isoflurane also inhibits not only presynaptic sodium channels but also action potential-evoked synaptic vesicle exocytosis by inhibiting presynaptic calcium influx into glutamatergic neurons to a greater degree than GABAergic neurons (Baumgart et al., 2015; Wang et al., 2020). As a result, not only was a lower dose of 4-AP required to elicit events in awake animals, but the area of ictal onset was smaller.

Propagation was also altered in awake mice who demonstrated contralateral propagation much more readily than under isoflurane anesthesia. Under awake conditions, contralateral spread occurred through two distinct mechanisms, either cross callosal or contiguous spread. The former is well-described *in vivo* but the latter mostly witnessed *in vitro*. Contiguous propagation likely relies on volume conduction through an electrical field (Zhang et al., 2014; Qiu et al., 2015; Shivacharan et al., 2019). During the ictal event, a large population of neurons fires synchronously creating an electrical field, which excites neighboring neurons and facilitates seizure propagation. Isoflurane anesthesia may blunt volume conduction as well as synaptic transmission thereby diminishing the variability of seizure propagation patterns that arise in awake animals. Likewise, isoflurane’s potentiation of GABA may limit contralateral spread.

In this study, we chose to image only the excitatory neurons during seizures. Activity in interneurons would not contribute to the optical calcium signal. Interneurons have been implicated in ictal onset, coordinating widespread synchronization of pyramidal cells and also in the restraint of ictal propagation, providing an inhibitory veto to lateral spread (Sessolo et al., 2015; de Curtis and Avoli, 2016; Liou et al., 2018). However, the primary drivers of the ictal event are bursting excitatory neurons and our imaging of this population of cells should provide a reasonable estimate of the ictal core as a basis for NVC analysis.

### Neurovascular Coupling and Epilepsy

Neurovascular Coupling-based hemodynamic responses [including CBF, CBV, and hemoglobin oxygenation (Girouard and Iadecola, 2006; Tian et al., 2010; Liu et al., 2013; Hall et al., 2014)] form the basis of a variety of brain imaging modalities used both in the laboratory and the clinic as surrogates for neuronal activity. Detailed understanding of the hemodynamic response function is required for correct interpretation of these signals, which can be altered by disease states such as epilepsy.

Studies of ictal events in both animals and humans have clearly demonstrated that seizures cause a massive increase in neuronal metabolism and a correspondingly large increase in CBF as a result of arteriolar vasodilation (Penfield, 1939; Dymond and Crandall, 1976; Horton et al., 1980; Siesjö et al., 1986; Weinand et al., 1994; Zhao et al., 2009, 2011). Previous research that has used the HbT or CBV signal as a mapping signal has shown that the signal may be highly localized depending on the species, the model, and the timing of signal capture (Zhao et al., 2007, 2009; Ma et al., 2013). Optical mapping of electrical activity in these prior studies was generally performed using LFP, ECoG, or VSD imaging, which reflects mostly subthreshold activity, or bulk loaded calcium dyes, which also stain glia and the neuropil, which is not specific for neuronal activity (Zhao et al., 2007, 2009; Ma et al., 2013, 2014a). In this study, the first to combine mesoscopic IOS with simultaneous genetically encoded calcium imaging, we have shown that in awake as well as anesthetized animals, the CBV (HbT) signal provides the highest resolution mapping signal of the hemodynamic responses. Since the calcium signal reflects mostly cells firing action potentials, which should provide a fairly accurate representation of the ictal core, the implication is that clinical imaging in humans for seizure localization should rely more on blood flow and volume than hemoglobin oxygenation (see below). Although SPECT and subtraction ictal SPECT co-registered with MRI (SISCOM) provide measures of blood flow, the image is static lacking sufficient temporal resolution, and the technique very difficult to perform due to its logistics (So, 2000; Van Paesschen, 2004; Habert and Huberfeld, 2008; Chiron, 2013).

Oxy- and deoxyhemoglobin (HbO and Hbr) have also been widely studied as sources of ictal mapping signals. During normal sensory processing, an early dip in hemoglobin oxygenation arising from a brief delay in vasodilation has been recorded with IOS and some fast fMRI studies (Frostig et al., 1990; Malonek and Grinvald, 1996; Logothetis et al., 1999; Vanzetta and Grinvald, 1999; Thompson et al., 2003). This early dip was shown to localize more precisely with neuronal activity during sensory processing in a number of early studies (Frostig et al., 1990; Malonek and Grinvald, 1996; Schwartz and Bonhoeffer, 2001; Ances, 2004; Sheth et al., 2004). A similar “epileptic dip,” often of much longer duration, was identified using IOS (Bahar et al., 2006; Zhao et al., 2009) and oxygen-sensitive electrodes (Zhao et al., 2009) in animal models under anesthesia and also in human spontaneous epilepsy with IOS (Zhao et al., 2007) and gold wire recordings of tissue oxygenation (Cooper et al., 1966; Dymond and Crandall, 1976). A similar dip is also present during single interictal spikes (Suh et al., 2005b; Ma et al., 2009b; Geneslaw et al., 2011). Of note, one study of chronic epilepsy in the MAM-pilocarpine model under anesthesia did not record an ictal increase in Hbr (Song et al., 2016). In the current study we have demonstrated that in awake animals, the epileptic dip does not provide a localizing signal and is inconsistently present during ictal events. We have also shown that in awake animals, the amplitude of the HbT signal increases compared with the anesthetized state and seems to provide sufficient oxygenated blood to prevent any consistent focal ischemia. For this same reason, the HbO signal is more diffuse and poorly localized. These findings should elicit caution in the interpretation of BOLD-fMRI for mapping ictal events since the BOLD signal is weighted heavily by the HbR signal.

### The Difference in the Hemodynamic Response Between Awake and Isoflurane-Anesthetized Condition

Different anesthetics work through distinct mechanisms and can have a variety of effects on NVC mechanisms leading to drastically different responses to the same experimental conditions (Lindauer et al., 1993; Bonvento et al., 1994; Gordon et al., 1995; Jones and Diamond, 1995; Linde et al., 1999; Kaisti et al., 2003; Oz et al., 2004; Austin et al., 2005; Du et al., 2009). Moreover, fluctuating levels of anesthesia can lead to intra-experimental variations in results (Masamoto and Kanno, 2012). For these reasons, studies of NVC performed under anesthesia may be misleading and awake data is required to accurately understand the hemodynamic response occurring in the unanesthetized state. Most anesthetics, including isoflurane, not only suppress neural activity but act as potent vasodilators through their actions on ATP-sensitive potassium channels and calcium currents in smooth muscle cells (Flynn et al., 1991, 1992; Van Aken and Van Hemelrijck, 1991). Laser-Doppler and two-photon studies of normal cortical processing show a reduction in both red blood cell velocity and concentration caused by isoflurane (Takuwa et al., 2012). Studies of normal sensory processing in rodents also report that hemodynamic signals evolve more slowly under anesthesia (Martin et al., 2006). Although our data indicate that anesthesia reduced the amplitude of the hemodynamic signals, the slope of the correlation between the calcium and the hemodynamic signals was similar in the awake and anesthetized states, indicating that both the calcium and hemodynamic signals were comparably dampened.

Our hypothesis regarding the mechanism for the amplification of the epileptic dip under isoflurane lies in the diminution of perfusion, reflected in the decrease in HbT amplitude. We tested this hypothesis with pharmacologic restriction of vascular reactivity and found that restraining vessel dilatation augmented the increase in Hbr also under awake conditions. Awake studies of sensory processing in rodents have also shown a reduction in the initial dip, although the opposite result was reported in non-human primates (Krasowski and Harrison, 2000; Shtoyerman et al., 2000; Martin et al., 2006). In contrast with the Hbr signal, we found that the HbO signal was amplified in the awake state as a result of an increase in CBF, CBV, and an exaggerated rise in the influx of oxygenated hemoglobin. Similar results have been reported in both animal and human studies (Lahti et al., 1998, 1999; Shtoyerman et al., 2000; Peeters et al., 2001; Berwick et al., 2002; Sicard et al., 2003). However, we find that the ictal HbO signal is not tightly linked to the area of the calcium signal, which localizes cells firing action potentials in the ictal core. Presumably, the BOLD signal would likewise overestimate the size of the seizure focus. On the other hand, the HbT signal was not only more highly localizing for the ictal core but the correlation coefficient was higher in the awake than in the anesthetized state. Not surprisingly, awake investigations of somatosensory processing in cats have also confirmed that the HbT signal provides a better map of neuronal activity in awake compared with anesthetized animals (Shtoyerman et al., 2000; Fukuda et al., 2005).

Another explanation for the spatial specificity of the HbT signal compared with oxygen-based signals could be the spatial origin of the different hemodynamic components. In wide-field optical imaging, it is possible to record hemodynamic changes from large and small arterioles, capillaries, parenchyma and small and large veins. The hemodynamic change varies based on the source of the signals (Ma et al., 2016). While the HbT signal is mostly related to distal perfusion from arteriolar dilatation related to increases in metabolism, oxygenation changes are impacted downstream of the metabolically active population of neurons, who alter hemoglobin oxygenation in the parenchyma and draining veins. The change of HbO and Hbr is thus determined by the local cerebral metabolic rate of oxygen (CMRO_2_) and the cerebral blood flow rate. In small and large veins, usually a very small HbT increase is recorded compared with a large-amplitude HbO increase and HbR decrease. Therefore, the BOLD signal, which is related to Hbr change, is usually located in the draining veins and should show a worse spatial overlap compared with the HbT signal.

In summary, in this first study to use wide-field mesoscopic genetically encoded calcium indicators to map ictal events for comparison with simultaneously recorded hemodynamic responses in awake behaving mice, we find that the removal of anesthesia increases the amplitude and spatial localization of the HbT response. Moreover, the spatial specificity of the HbO response diminishes while the Hbr signal becomes inconsistent and unreliable. Technical advances in chronic mapping of either calcium dynamics or HbT in human patients may be useful clinically to map ictal onset and propagation.

## Data Availability Statement

The raw data supporting the conclusions of this article will be made available by the authors, without undue reservation.

## Ethics Statement

The animal study was reviewed and approved by Weill Cornell Medical College Animal Care and Use Committee.

## Author Contributions

HM, TS, and WL designed the study. FY, JL, YS, MZ, JN, PL, and DL acquired and analyzed the data. FY, HM, and TS wrote the manuscript. All authors contributed to the article and approved the submitted version.

## Conflict of Interest

The authors declare that the research was conducted in the absence of any commercial or financial relationships that could be construed as a potential conflict of interest.

## Publisher’s Note

All claims expressed in this article are solely those of the authors and do not necessarily represent those of their affiliated organizations, or those of the publisher, the editors and the reviewers. Any product that may be evaluated in this article, or claim that may be made by its manufacturer, is not guaranteed or endorsed by the publisher.
