# Supplementary figures and images for: Mesoscopic Mapping of Ictal Neurovascular Coupling in Awake Behaving Mice Using Optical Spectroscopy and Genetically Encoded Calcium Indicators
Source: Front Neurosci. 2021 Jul 23;15:704834. doi: 10.3389/fnins.2021.704834 (PMC8343016; doi:10.3389/fnins.2021.704834)

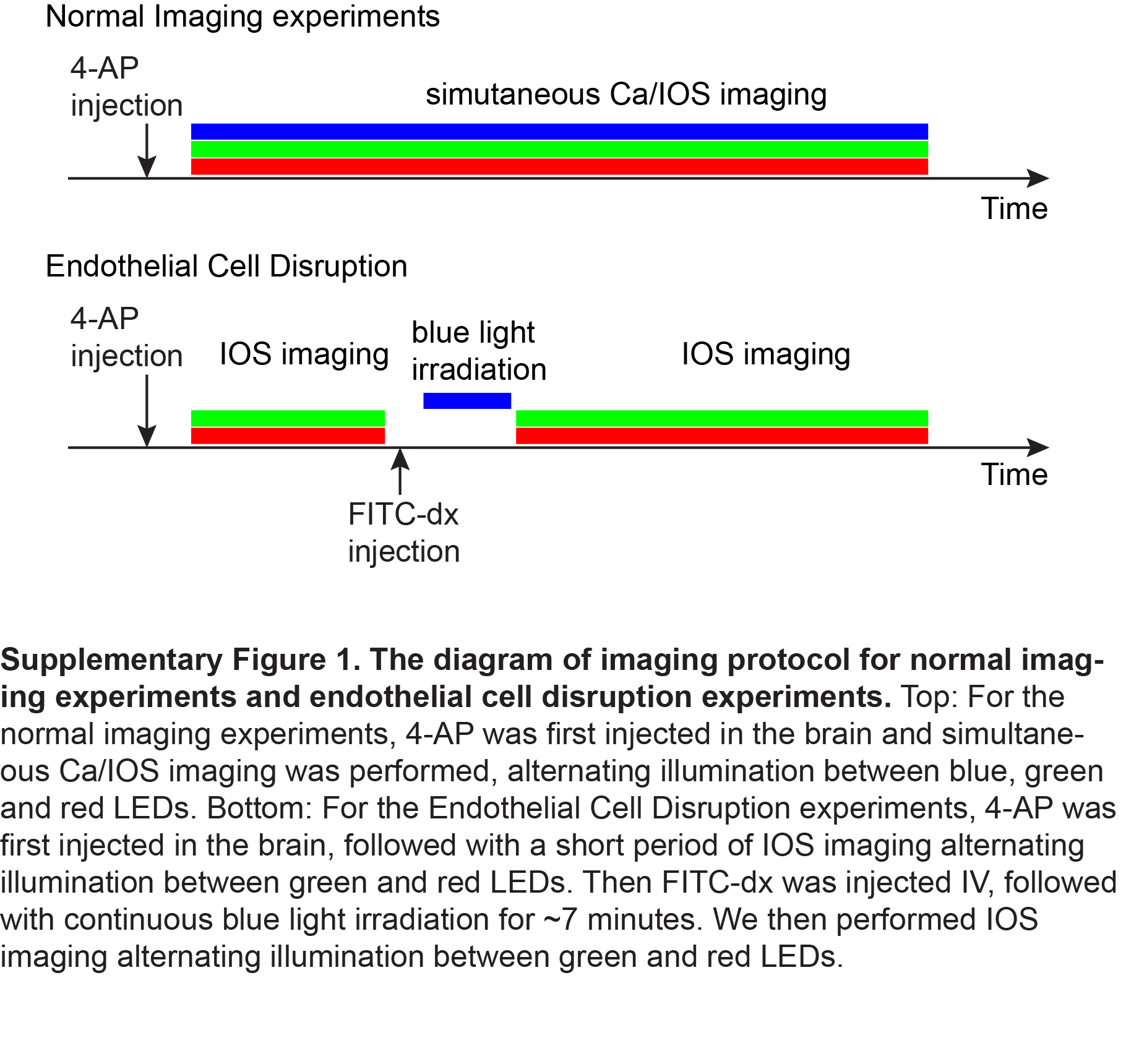

Supplement: Supplementary file 1 [file Image_1.JPEG]

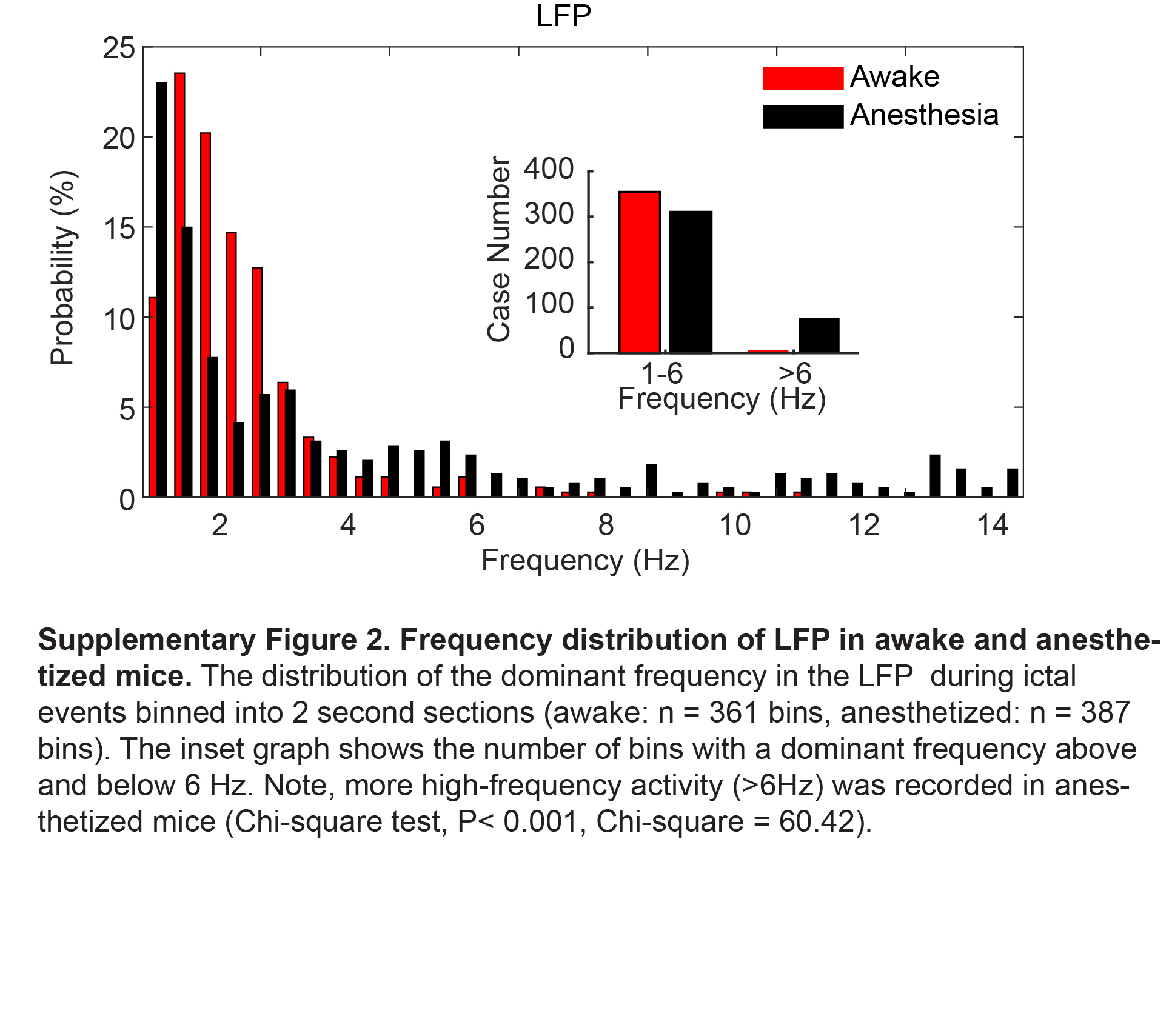

Supplement: Supplementary file 2 [file Image_2.JPEG]
